# Direct and Rapid Detection of *Mycoplasma bovis* in Bovine Milk Samples by Recombinase Polymerase Amplification Assays

**DOI:** 10.3389/fcimb.2021.639083

**Published:** 2021-02-25

**Authors:** Ruiwen Li, Jinfeng Wang, Xiaoxia Sun, Libing Liu, Jianchang Wang, Wanzhe Yuan

**Affiliations:** ^1^ College of Veterinary Medicine, Hebei Agricultural University, Baoding, China; ^2^ Food Microbiology and Animal Quarantine Laboratory, Technology Center of Shijiazhuang Customs District, Shijiazhuang, China

**Keywords:** *Mycoplasma bovis*, *uvrC* gene, real-time RPA, LFS RPA, isothermal amplification

## Abstract

This study aimed to detetct *Mycoplasma bovis* (*M. bovis*) in bovine milk quickly and directly by developing and validating isothermal recombinase polymerase amplification (RPA) assays. Targeting the *uvrC* gene of *M. bovis*, an RPA assay based on the fluorescence monitoring (real-time RPA) and an RPA assay combined with a lateral flow strip (LFS RPA) were conducted. It took 20 min for the real-time RPA to finish in a Genie III at 39°C, and 15 min were required to perform the LFS RPA in an incubator block at 39°C, followed by the visualization of the products on the lateral flow strip within 5 min. Both of the two assays showed high specificity for *M. bovis* without any cross-reaction with the other tested pathogens. With the standard recombinant plasmid pMbovis-uvrC serving as a template, both RPA assays had a limit of detcion of 1.0 × 10^1^ copies per reaction, equivalent to that of a real-time PCR assay. In the 65 milk samples collected from cattle with mastitis, the *M. bovis* genomic DNA was detected in 24 samples by both the real-time RPA and the LFS RPA assays. The developed RPA assays could detect *M. bovis* in bovine milk in an efficient, convenient, and credible manner as attractive and promising tools, and the assays would be helpful in the rapid response to *M. bovis* infection causing bovine mastitis.

## Introduction

As a major etiological agent of bovine mycoplasmosis globally, *Mycoplasma bovis* (*M. bovis*) causes various clinical symptoms in cattle, including pneumonia, arthritis, and mastitis (Nicholas and Ayling, 2003). Consequently, in addition to being recognized as a major pathogen in bovine respiratory disease complex (BRDC), *M. bovis* has also been found to cause cattle mastitis (Nicholas, 2011; Gioia et al., 2016). More importantly, infections with *M. bovis* cause considerable economic loss in the beef and dairy cattle industry, approximately 150 million euros across Europe as well as over $100 million per year in the United States (Nicholas and Ayling, 2003). However, it is difficult for *M. bovis* to be eradicated from a farm after an outbreak, and one infected cattle could be an infection source for months or even years (Burki et al., 2015).

As mentioned, rapid and accurate detection of *M. bovis* is imperative for effective prevention and control of the disease. Although bacteriological culture is considered to be the gold standard for the diagnosis of infection, routine diagnosis is not prioritized in practice. The culture method tends to be laborious, time-consuming, and lacks sensitivity and specificity owing to the fastidious nature of *M. bovis*, overgrowth of other contaminant bacteria, and subsequent difficulties in species identification (Khodakaram-Tafti and Lopez, 2004; Caswell and Archambault, 2007). Serological methods are typically used as a herd-level disease diagnostic test, and a variety of commercial *M. bovis* ELISA tests have been developed to detect antibodies in the milk and serum (Heller et al., 1993; Le et al., 2002; Nicholas and Ayling, 2003). However, they are not ideally suited for *M. bovis* infection investigations with individual animals, as a misdiagnosis may occur due to the delayed seroconversion after natural infection (Calcutt et al., 2018). Moreover, the high level of seroprevalence in many cattle herds restricts their routine use for diagnosis (Calcutt et al., 2018).

In efforts to avoid such disadvantages, diverse nucleic acid amplification approaches have been reported to sensitively, specifically, and immediately detect *M. bovis* from clinical samples, including milk (Clothier et al., 2010; Higa et al., 2016; Ashraf et al., 2018; Zhao et al., 2018). Dubbed the prime choice for molecular detection, PCR assays have been well established in the clinical diagnosis of cattle herds with BRDC and/or mastitis (Parker et al., 2018). However, compared with PCR assays, isothermal nucleic acid amplification assays, which are comparable to PCR and have a faster time-to-result in many cases, are more approporiate for the small-footprint devices in low-resource settings (Craw and Balachandran, 2012).

Among the recent isothermal nucleic acid amplification technologies, RPA has come into the spotlight because of its simplicity to design and optimize and its speed to obtain results. Thus, RPA technology has been widely utilized to explore different pathogens, as it tends to be fast, easy, and accurate (Piepenburg et al., 2006; Daher et al., 2016). In this paper, an exo probe-based real-time RPA and an nfo probe-based LFS RPA assays were developed and analyzed for their sensitivity and specificity for the direct detection of *M. bovis* in milk samples from cattle with mastitis.

## Materials and Methods

### Bacteria Strains and Clinical Samples

M. bovis (strain PG45), Mycoplasma agalactiae (strain PG2), Mycoplasma ovipneumoniae (strain Y98), Mycoplasma hyopneumoniae (strain 168), Mycoplasma capricolum subsp. capripneumoniae (strain F38), Pasteurella multocida (strain F91G3), Staphylococcus aureus (ATCC 6538), and Pseudomonas aeruginosa (ATCC 9027) were reserved in our laboratory.

Sixty-five individual bovine milk samples were collected from eight different dairy farms in Baoding and Hengshui, Hebei Province, from July 2018 to December 2020. The samples were all gathered from cattle with mastitis.

### DNA Extraction

The mycoplasma and bacterial genomic DNA were extracted with the TIANamp Bacteria DNA kit (Tiangen, Beijing, China), following the manufacturer’s instructions. First, 1 ml of each milk sample was centrifuged at 12,000 g for 10 min at 4°C, the supernatant containing the fat and excess liquid was removed, and the pellet was washed twice with phosphate-buffered saline (PBS, pH 7.4). The washed pellet was resuspended in 200 μl of PBS and then the DNA was extracted with the TIANamp Bacteria DNA kit. The extracted DNA was eluted in 50 μl of nuclease-free water, and the DNA was quantified with an ND-2000c spectrophotometer (NanoDrop, Wilmington, USA) and stored at −80°C until use.

### Generation of Standard DNA

Aimed to generate a *M. bovis*-standard DNA for the RPA assays, a PCR product with 1,908 bp covering the region of interest of *uvrC* gene, was amplified from the *M. bovis* DNA using uvrC-F and uvrC-R as primers (**Table 1**) and cloned into the pMD19-T (Takara, Dalian, China) for standards. The generating plasmid, pMbovis-uvrC, was transformed into *Escherichia coli* DH5α cells and the positive clones were identified by sequencing with M13 primers (Invitrogen^®^, Carlsbad, CA, USA). pMbovis-uvrC was purified with the SanPrep Plasmid MiniPrep Kit (Sangon Biotech, Shanghai, China) and quantified with a ND-2000c spectrophotometer. The copy number of DNA molecules was calculated according to the formula as follows: amount (copies/μl) = [DNA concentration (g/μl)/(plasmid length in base pairs × 660)] × 6.02 × 10^23^. Aliquots of the standard DNA were prepared in 10-fold serial dilutions from 1.0 × 10^7^ to 1.0 × 10^0^ copies/μl in nuclease-free water and stored at −80°C until use.

**Table 1:** Sequences of the primers and probes used in this study.

Assay	Primers and probes	Sequence 5´-3´	Amplicon size (bp)	Reference
Real-time RPA	uvrC-exo-F	GAGTTTCACAAAACCAAAGCCTTAATTGACCT	180	This study
	uvrC-exo-R	TCCTTTTATGTTTCTTAGTTTGCCTTCTAGTG		
	uvrC-exo-P	CTTAGTTCAAATTCAAGTTGACCGG (FAM-dT) (THF)(BHQ1-dT) GCAAAGTCGCACTT-C3-spacer		
LFS RPA		GAGTTTCACAAAACCAAAGCCTTAATTGACCT	180	This study
		biotin-TCCTTTTATGTTTCTTAGTTTGCCTTCTAGTG		
		FAM-CTTAGTTCAAATTCAAGTTGACCGGT(THF)TGCAAAGTCGCACTT-C3-spacer		
Real-time PCR	Mbov F2024	TCTAATTTTTTCATCATCGCTAATGC	112	Clothier et al., 2010
	Mbov R2135	TCAGGCCTTTGCTACAATGAAC		
	Mbov uvrC	FAM-AACTGCATCATATCACATACT-MGB		
PCR	uvrC-F	GCAAAGAATTTACGCAAGAG	1908	This study
	uvrC-F	GACTTTGAAATAACTAGACCAGT		

### RPA Primers and Probes

Nucleotide sequence data for different *M. bovis* strains available in GenBank were aligned to identify the conserved regions in the *uvrC* gene, which was determined as the amplification target for RPA. Basing on the reference sequences of *M. bovis* (Acession number: AF003959, KX772801, KX772803, CP045797, KU168366, KP099619), the primers, exo probe and LF probe were designed following the RPA manufacturer guidelines (TwistDx. Cambridge, UK). Primers and probes are presented in **Table 1** and were synthesized by Sangon Biotech Co., Shanghai, China.

### Real-Time RPA Assay

A commercial ZC BioScience™ exo kit (ZC BioScience, Hangzhou, China) was employed in the *M. bovis* real-time RPA assay. The reaction volume was 50 μl including 40.9 μl of Buffer A (rehydration buffer), 2.0 μl of each RPA primer (uvrC-exo-F and uvrC-exo-R, 10 μmol/L), 0.6 μl of exo probe (uvrC-exo-P, 10 μmol/L), and 2.5 μl of Buffer B (magnesium acetate, 280 mmol/L). Additionally, 1 μl of bacterial genomic DNA or standard DNA was used for the specificity and sensitivity analysis, while 2 μl of sample DNA was used for the clinical sample diagnosis. Real-time RPA reactions were performed at 39°C for 20 min in a Genie III (OptiGene Limited, West Sussex, UK).

### LFS RPA Assay

A commercial TwistAmp™ nfo kit (TwistDX, Cambridge, UK) and lateral flow strip (USTAR, Hangzhou, China) were utilized in the *M. bovis* LFS RPA assay. The reaction volume was 50 μl including 29.5 μl of rehydration buffer, 2.1 μl of each RPA primer (uvrC-nfo-F and uvrC-nfo-R, 10 μmol/L), 0.6 μl of exo probe (uvrC-nfo-P, 10 μmol/L), and 2.5 μl of magnesium acetate (280 mmol/L). In addition, 1 μl of bacterial genomic DNA or standard DNA was used for the specific and sensitive analysis, while 2 μl of sample DNA was used for the clinical sample diagnosis. The LFS RPA reactions were incubated in an incubator block at 39°C for 5, 10, 15, and 20 min. The lateral flow strips were used to recognize the amplicons dual-labeled with FAM and biotin. The LFS RPA products were identified visually by using lateral flow strips according to the manufacturer’s instructions.

### Analytical Specificity and Sensitivity Analysis

The specificity of the developed real-time RPA and LFS RPA assays was assessed using the genomic DNA of a panel of pathogens, including *M. bovis*, *M. agalactiae*, *M. ovipneumoniae*, *M. hyopneumoniae*, *M. capricolum subsp. capripneumoniae*, *P. multocida*, *S. aureus*, and *P. aeruginosa*. Five of these were closely related Mycoplasma species, and the other three could potentially be associated with mastitis in cattle. The assays were conducted independently in triplicate.

Aliquots of the *M. bovis* standard DNA ranging from 1.0 × 10^7^ to 1.0 × 10^0^ copies/μl were used to analyze the RPA analytical sensitivity. One microliter of each dilution was amplified by both RPA assays, and the limit of detection (LOD) was determined as the highest dilution of the virus detectable by the assays. The real-time RPA was additionally tested using the standard DNA in eight replicates, the threshold time was plotted against the molecules identified, and a semilog regression was calculated by Prism software 5.0 (GraphPad Software Inc., San Diego, CA, USA). Furthermore, in the LFS RPA, three independent reactions proceeded separately.

### Validation With Clinical Samples

The developed assays were directly applied to DNA extracted from 65 bovine milk samples to confirm the applicability of the *M. bovis*-specific real-time RPA and LFS RPA assays in clinical diagnosis,. Then, the results were compared with those obtained with real-time PCR described previously (Clothier et al., 2010), which was run in parallel for the above clinical samples.

## Results

### Performance of the Real-Time RPA Assay

**Figure 1:**
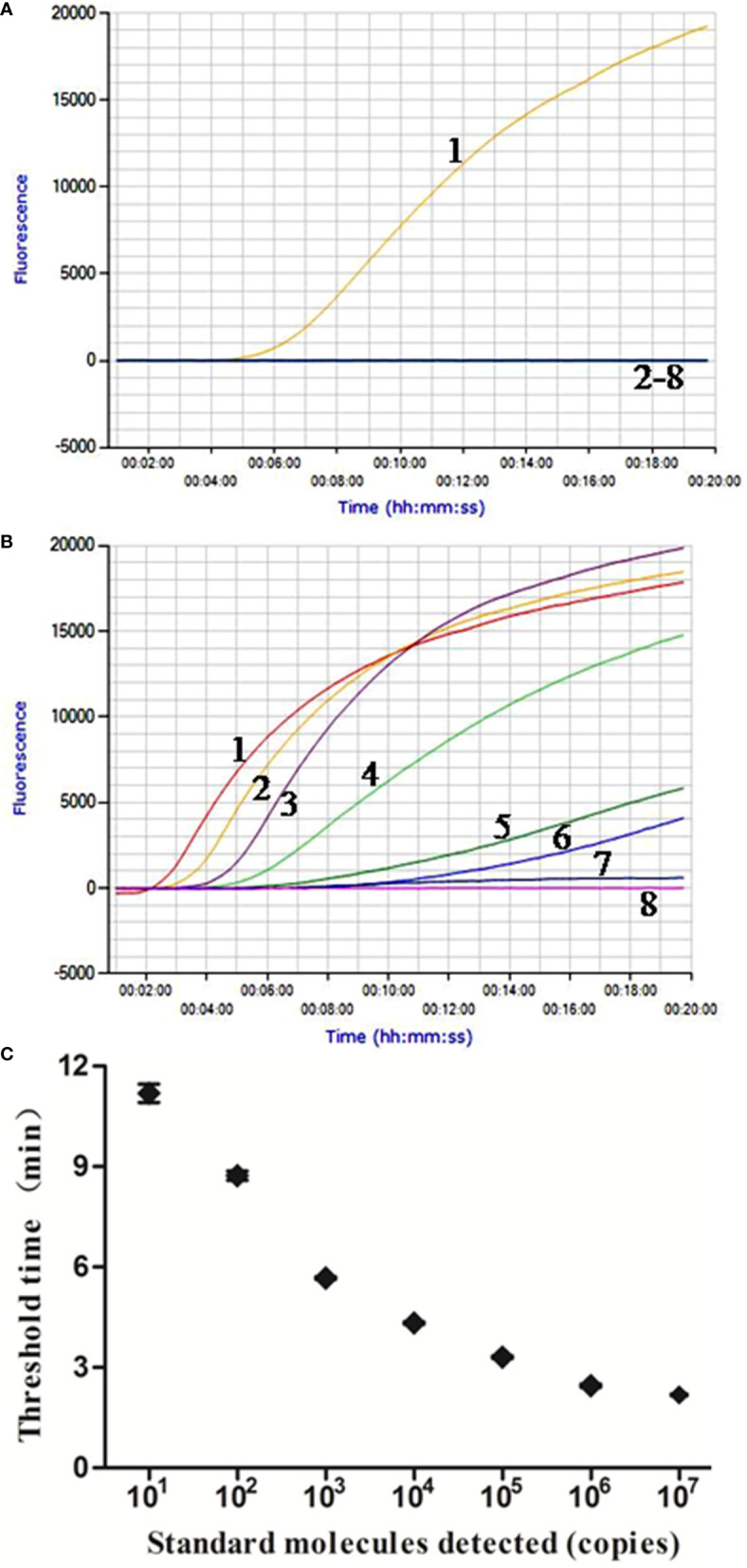
Performance of *M. bovis* real-time RPA assay. **(A)** Analytical specificity of the real-time RPA assay. Only the *M. bovis* was amplified and there were no cross-reactions with other pathogens tested. Line 1, *M. bovis*; line 2, *M. agalactiae*; line 3, *M. ovipneumoniae*; line 4, *M. hyopneumoniae*; line 5, *M. capricolum subsp. capripneumoniae*; line 6, *P. multocida*; line 7, *S. aureus*; line 8, *P. aeruginosa*. **(B)** Fluorescence development over time using a dilution range of 1.0 × 10^7^–1.0 × 10^0^ copies of *M. bovis* standard recombinant plasmid. Line 1, 1.0 × 10^7^ copies; line 2, 1.0 × 10^6^ copies; line 3, 1.0 × 10^5^ copies; line 4, 1.0 × 10^4^ copies; line 5, 1.0 × 10^3^ copies; line 6, 1.0 × 10^2^ copies; line 7, 1.0 × 10^1^ copies; line 8, 1.0 × 10^0^ copies. **(C)** Reproducibility of *M. bovis* real-time RPA assay. The data collected from *M. bovis* real-time RPA tests and the semi-log regression was calculated using Prism Software. The run time of the real-time RPA was between 2 min and 12 min for 1.0 × 10^7^ and 1.0 × 10^1^ copies.

In the analytical specificity analysis, only the *M. bovis* DNA was amplified with the development of a typical fluorescence curve, and none of the other pathogens were amplified (**Figure 1A**), suggesting that the real-time RPA assay was highly specific to *M. bovis*. Similar results were observed in three repeats, demonstrating the good repeatability of the assays.

The real-time RPA assay was conducted eight times, in which 1.0 × 10^7^–1.0 × 10^1^ copies of standard plasmid were detected in 8/8 runs, and 1.0 × 10^0^, 0/8 (**Figure 1B**). From these results, what have been exhibited is the LOD for the real-time RPA was 1.0 × 10^1^ copies/reaction. Moreover, the dynamic detection range of the real-time RPA spans seven logs ranging from seven to one log copies per reaction, with the corresponding threshold time ranging from 2 min at 1.0 × 10^7^ copies/reaction to 12 min at 1.0 × 10^1^ copies/reaction, which revealed that the *M. bovis* real-time RPA assay has a wide dynamic range to detect the target DNA (**Figure 1C**).

### Performance of the LFS RPA Assay

**Figure 2:**
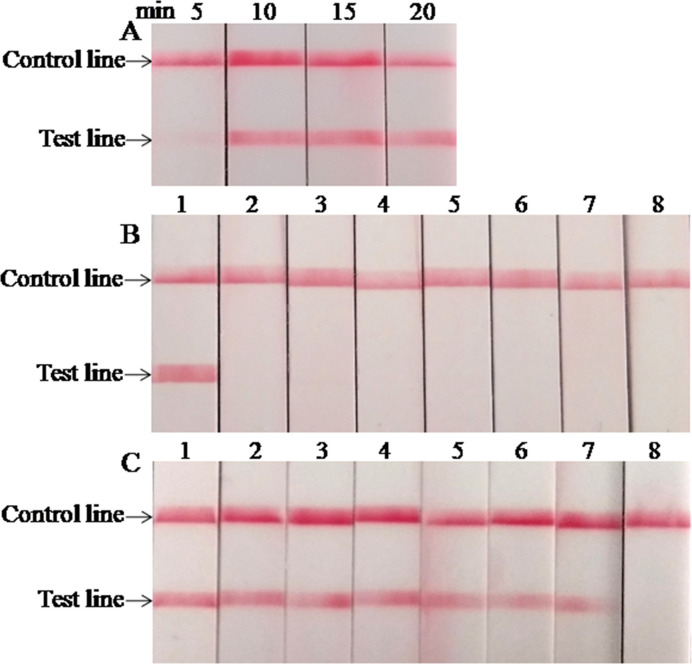
Performance of *M. bovis* LFS RPA assay. **(A)** Optimization of LFS RPA reaction time. The test line was clearly visible when the amplification time was longer than 10 min. **(B)** Analytical specificity of the LFS RPA assay. Only the *M. bovis* was amplified, but not other pathogens tested. Line 1, *M. bovis*; line 2, *M. agalactiae*; line 3, *M. ovipneumoniae*; line 4, *M. hyopneumoniae*; line 5, *M. capricolum subsp. capripneumoniae*; line 6, *P. multocida*; line 7, *S. aureus*; line 8, *P. aeruginosa*. **(C)** Analytical sensitivity of the LFS RPA assay. Line 1, 1.0 × 10^7^ copies; line 2, 1.0 × 10^6^ copies; line 3, 1.0 × 10^5^ copies; line 4, 1.0 × 10^4^ copies; line 5, 1.0 × 10^3^ copies; line 6, 1.0 × 10^2^ copies; line 7, 1.0 × 10^1^ copies; line 8, 1.0 × 10^0^ copies.

The optimal reaction time of the LFS RPA assay was evaluated by testing the results at 5, 10, 15, and 20 min with 1.0 × 10^5^ copies standard plasmids as the template, and the results are presented in **Figure 2A**. A very weak red band was observed after 5 min of incubation, and no distinct differences were observed among the products after 10, 15, and 20 min of incubation. Similar results were obtained from three repeats. Based on the above results, 15 min was selected as the best incubation time for the *M. bovis* LFS RPA assay.

When the analytical specificity analysis was conducted, the red band was only observed in the test line on the strip when the DNA of *M. bovis* was used as the template (**Figure 2B**), and the same results were seen in three independent reactions that the same results were received. As mentioned above, these results revealed that the LFS RPA assay was highly specific for detecting *M. bovis* and showed no cross-reactions with the other pathogens tested. As the analytical sensitivity analysis proceeded, red bands could be observed in the test line on the strips with 1.0 × 10^7^–1.0 × 10^1^ copies of standard plasmid serving as the template (**Figure 2C**), and all three independent reactions showed identical results. Thus, the LOD of the LFS RPA was 1.0 × 10^1^ copies/reaction.

### Validation of the RPA Assays on Clinical Samples

**Figure 3:**
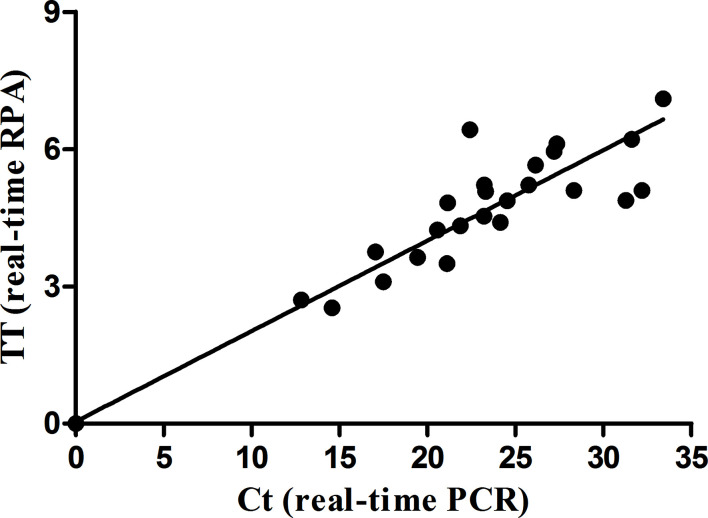
Comparison between performances of the real-time RPA and real-time PCR on the milk samples. DNA extracts of the positive milk samples were screened. Linear regression analysis of real-time RPA threshold time (TT) values (y axis) and real-time PCR cycle threshold (Ct) values (x axis) were determined by Prism software, and the R^2^ value was 0.951.

The real-time RPA, LFS RPA, and real-time PCR all had identical results for the 65 milk samples from eight farms, in which 24 samples from four farms tested positive for *M. bovis* (**Table 2**). Using the real-time PCR as the reference, the diagnostic specificity (DSp) and diagnostic sensitivity (DSe) of the real-time RPA and LFS RPA assays were 100%. The threshold time (TT) and cycle threshold (Ct) values of the real-time RPA and real-time PCR were good at an R^2^ value of 0.951 (**Figure 3**).

**Table 2:** Detection results of *M. bovis* in milk samples from cattle with mastitis in the developed real-time RPA, LFS RPA, and real-time PCR assays.

Origin	Location	Number	Real-time RPA		LFS RPA		Real-time PCR	
			P	N	P	N	P	N
Farm 1	Baoding	17	10	7	10	7	10	7
Farm 2	Baoding	2	2	0	2	0	2	0
Farm 3	Baoding	11	5	6	5	6	5	6
Farm 4	Baoding	6	0	6	0	6	0	6
Farm 5	Hengshui	2	0	2	0	2	0	2
Farm 6	Hengshui	7	0	7	0	7	0	7
Farm 7	Hengshui	11	7	4	7	4	7	4
Farm 8	Hengshui	9	0	9	0	9	0	9
	T	65	24	41	24	41	24	41

P, positive; N, negative; T, total.

## Discussion

*M. bovis* is the most prevalent agent of mycoplasma mastitis in dairy cattle (Gioia et al., 2016). It can survive in the milk of asymptomatically infected and clinically healthy cows, and ingestion of milk from cows with mastitis is one of the primary modes of transmission (Maunsell et al., 2011). Both bovine individual and bulk tank milk samples have been applied to recognize *M. bovis* in daily surveillance and eradication efforts (Passchyn et al., 2012; Pinho et al., 2013). Concerning the *uvrC* gene, this paper proposed and demonstrated that real-time RPA and LFS RPA assays could directly identify *M. bovis* in milk samples, and the tests were proven to be rapid, sensitive, and specific.

Selecting the target gene is critical for the application of nucleic acid amplification. A series of PCR and LAMP assays targeting various genes, such as *uvrC*, *oppD/F*, *polC*, gyrB, and *16S rRNA*, have been conducted for the species-specific detection of *M. bovis* in diverse clinical samples from cattle herds with BRDC and/or mastitis (Clothier et al., 2010; Rossetti et al., 2010; Ashraf et al., 2018). A study indicated that most PCR assays targeting different genes performed comparatively (Wisselink et al., 2019). Nevertheless, all the sequences of the 16S rRNA genes of *M. bovis* and *M. agalactiae* are extremely similar (>99.8%) (Pettersson et al., 1996; Konigsson et al., 2002), the available *M. bovis* PCR and LAMP assays targeting the *16S rRNA* gene exhibited cross-reactivity with *M. agalactiae* (Ashraf et al., 2018; Wisselink et al., 2019). The *uvrC* gene is a highly conserved housekeeping gene specific for each of *Mycoplasma* species that is highly stable within a species, and it differs considerably between the two phylogenetically closely related *Mycoplasma* species, *M. bovis* and *M. agalactiae* (Subramaniam et al., 1998; Konigsson et al., 2002). Several previous studies have revealed that the *uvrC* gene is universally deemed to be the preferred target for *M. bovis* in the nucleic acid amplification assays (Clothier et al., 2010; Ashraf et al., 2018; Zhao et al., 2018; Wisselink et al., 2019). As a result, the *M. bovis* RPA primers and probes were designed to target the conserved region of the *uvrC* gene in this study. Through *in silico* analysis, there was no mismatch in the primers and probes with the currently circulating strains available in GenBank. For the specificity analysis, both the real-time RPA and the LFS RPA could only amplify the genomic DNA of *M. bovis* but not the other mycoplasmas, including *M. agalactiae*. With the recombinant plasmid being the standard, the LOD of both RPA assays was 1.0 × 10^1^ copies per reaction. Unfortunately, only one *M. bovis* strain and one *M. agalactiae* stain were considered in the analysis, which may be a shortcoming of this study. The developed assays should be further confirmed by testing more *M. bovis* and *M. agalactiae* DNA extracts in the future.

Directly detecting *M. bovis* in milk samples has the potential to be of great significance for the control of *M. bovis*-causing bovine mastitis. Fortunately, the developed real-time RPA and LFS RPA assays could detect the *M. bovis* in a direct and efficient way from clinical bovine milk samples. In this study, the *M. bovis* positive rate at the farm level and at the individual level reached 50.0% (4/8) and 36.92% (24/65), respectively. Compared with a real-time PCR assay, the real-time RPA and LFS RPA assays showed DSp and Dse values of 100%. The RPA assays showed positive results within 20 min, demonstrating Ct values varying from 12.83 to 33.40 for the real-time PCR. The diagnostic performances of the developed RPA assays were the same as that of the real-time PCR assay, while the RPA assays demonstrated two distinct merits, rapidness and convenience. Although the above results are inspiring, RPA assays still require further validation by testing additional types of *M. bovis* DNA-positive clinical samples, such as nasal swabs and lungs. Overall, our results demonstrated that the performance of the RPA assays was comparable to that of real-time PCR, but the RPA assays were relatively faster.

Considered the prime assay in the realm of molecular detection, the PCR assays are, however, limited in underequipped laboratories and at point-of-need (PON) diagnosis owing to the demands of expensive thermocyclers and centralized laboratory facilities (Clothier et al., 2010; Rossetti et al., 2010). The recently developed LAMP assays do not require a specialized instrument, but the reaction time is 60 or 120 min (Higa et al., 2016; Ashraf et al., 2018). In this study, the developed real-time RPA assay and LFS RPA assay were performed on the portable tube scanner Genie III and in a metal bath incubator, respectively. Both of these devices are portable and can be charged by a battery, allowing them to work for an entire day with no need for an electrical outlet. Combined with the time needed for DNA extraction, the developed RPA assays require less than 50 min to obtain results. Moreover, RPA reagents are cold chain independent and RPA is tolerant to common PCR inhibitors (Daher et al., 2016; Lillis et al., 2016). These advantages make the developed RPA assays perfect for detecting *M. bovis* in the field.

Similar to the PCR and LAMP assays, DNA extraction by commercial nucleic acid extraction kits is still necessary for the RPA assays developed in this study. Currently, numerous simple and rapid nucleic acid extraction methods that do not require complex instruments are being evaluated in our laboratory, including the innuPREP MP basic kit A (Jena Analytik, Jena, Germany), Punch-it™ NA-Sample Kit (NanoHelix, Daejeon, South Korea), and other commercial reagents. Combining the DNA extracted by those simple methods with comparable performance to routine commercial nucleic acid extraction kits has the potential to allow the recentlyl developed RPA assays for *M. bovis* to be applied in the field.

In summary, the developed *M. bovis* real-time RPA and LFS RPA assays could be performed in the laboratory as routine diagnostic assays, and they have substantial potential as uncomplicated, rapid, and reliable methods for directly detecting *M. bovis* in bovine milk on farm.

## Data Availability Statement

The original contributions presented in the study are included in the article/supplementary material. Further inquiries can be directed to the corresponding authors.

## Author Contributions

Among the authors, RL and JFW were responsible for sampling, sample testing, and writing the paper. XS and LL were responsible for data statistics and analysis. JCW and WY were responsible for editing the paper. All authors contributed to the article and approved the submitted version.

## Funding

This work was supported by the Project for Key Common Technologies for High Quality Agricultural Development of Hebei Province (19226636D), the Research Programm of General Administration of Customs (2020HK170).

## Conflict of Interest

The authors declare that the research was conducted in the absence of any commercial or financial relationships that could be construed as a potential conflict of interest.
